# Phase Behavior of Ion-Containing Polymers in Polar Solvents: Predictions from a Liquid-State Theory with Local Short-Range Interactions

**DOI:** 10.3390/polym14204421

**Published:** 2022-10-19

**Authors:** Yanwei Wang, Qiyuan Qiu, Arailym Yedilbayeva, Diana Kairula, Liang Dai

**Affiliations:** 1 Department of Chemical & Materials Engineering, School of Engineering and Digital Sciences, Nazarbayev University, Nur-Sultan 010000, Kazakhstan; 2 Laboratory of Computational Materials Science for Energy Applications, Center for Energy and Advanced Materials Science, National Laboratory Astana, Nur-Sultan 010000, Kazakhstan; 3 Department of Physics, City University of Hong Kong, Kowloon, Hong Kong 999077, China; 4 Shenzhen Research Institute, City University of Hong Kong, Shenzhen 518057, China

**Keywords:** charged polymers, polymer solutions, electrostatic interactions, counterion, water-soluble polymers, theory

## Abstract

The thermodynamic phase behavior of charged polymers is a crucial property underlying their role in biology and various industrial applications. A complete understanding of the phase behaviors of such polymer solutions remains challenging due to the multi-component nature of the system and the delicate interplay among various factors, including the translational entropy of each component, excluded volume interactions, chain connectivity, electrostatic interactions, and other specific interactions. In this work, the phase behavior of partially charged ion-containing polymers in polar solvents is studied by further developing a liquid-state (LS) theory with local shortrange interactions. This work is based on the LS theory developed for fully-charged polyelectrolyte solutions. Specific interactions between charged groups of the polymer and counterions, between neutral segments of the polymer, and between charged segments of the polymer are incorporated into the LS theory by an extra Helmholtz free energy from the perturbed-chain statistical associating fluid theory (PC-SAFT). The influence of the sequence structure of the partially charged polymer is modeled by the number of connections between bonded segments. The effects of chain length, charge fraction, counterion valency, and specific short-range interactions are explored. A computational App for salt-free polymer solutions is developed and presented, which allows easy computation of the binodal curve and critical point by specifying values for the relevant model parameters.

## 1. Introduction

Charged polymers [1,2,3], or according to the “Terminology of polymers containing ionizable or ionic groups and of polymers containing ions (IUPAC Recommendations 2006)” [4], ion-containing polymers or ionic polymers, are macromolecules containing ionic or ionizable groups, or both, irrespective of their nature, content, and location. In the category of ionic polymers, there are anionic polymers, cationic polymers, and ampholytic polymers [5]. If a substantial portion of the constitutional units contains ionic or ionizable groups, or both, such ion-containing polymers are often called polyelectrolytes [6,7]. According to Hoagland [7], charged polymers possessing only a low density of charged units along their backbones, with the fraction of these units typically less than about 15% on a mole basis, are often referred to as ionomers. The ionic groups that endow ionic polymers are no different than those found in small organic molecules bearing charges. The list of anionic groups includes, e.g., sulfate, phosphate, sulfonate, and carboxyl groups. The list of cationic groups includes, e.g., protonated ammonium, quaternized ammonium, sulfonium, and phosphonium groups. For summaries of chemical structures of those ionizable or ionic groups, interested readers are directed to the reviews by Hoagland [7], Mecerreyes [8], and more recently Kocak et al. [9] and Ofridam et al. [10].

Charged polymers are ubiquitous throughout nature and have myriad technological applications [7,8,9,10,11,12,13,14,15,16]. Most of the water-soluble polymers, either natural or synthetic, are charged, and they have a wide range of applications in various industrial sectors, including the pharmaceutical and biomedical industries [12], the oil and gas industries [17,18,19,20], construction chemicals [13], coatings, inks, flocculants, papers, agrochemicals (or agrichemicals), adhesives, foodstuffs, pharmaceuticals, cosmetics, and personal care products [21]. When water-soluble polymers containing ionizable groups come into contact with water, which is a polar solvent, they dissolve and release “counterions” into their surroundings. Counterions have more freedom to move around within the solvent domain, although long-range electrostatic interactions may limit the extent to which they do so [22,23]. Even in liquid water, which possesses a relatively high dielectric constant, electrostatic forces strongly oppose the dissociation and physical separation of unlike charges [7]. Thus, a diffuse cloud of small counterions closely surrounds a dissolved charged polymer. Counterions in the diffuse cloud, as well as other small ions of an added electrolyte, screen electrostatic interactions. Thus, adding an electrolyte to a polyelectrolyte solution contracts the counterion cloud; at sufficiently high electrolyte concentrations, the cloud’s shrinkage onto the chain transforms many polyelectrolyte properties to those of a neutral polymer [24]. Conversely, with no added electrolyte, and thus only liberated counterions present (a special condition termed “salt-free”), distinctive polyelectrolyte behaviors are strongly magnified [7].

The phase behavior of polymer solutions and blends, including those of charged polymers, is a fundamental problem in polymer science [25,26]. Understanding of the phase behavior of polymer solutions and blends has grown steadily following the development of the Flory–Huggins theory in 1942 [27,28,29,30,31]. For neutral polymers dissolved in poor solvents, when the polymer concentration is increased, the polymers tend to aggregate, and beyond a certain concentration two phases appear, one of dilute solution and another of concentration solution. This phenomenon is called “phase separation”. When studying the thermodynamics of polymers, the binodal curve (or coexistence curve) [32] denotes the temperature and composition conditions at which two distinct phases may coexist. Equivalently, it is the boundary between the set of conditions in which it is thermodynamically favorable for the system to be fully mixed and the set of conditions in which it is thermodynamically favorable for it to phase separate. For solutions of simple neutral polymers, the binodal curves can often be predicted by theories of the Flory–Huggins type [25,26]. A number of studies [33,34,35,36,37,38,39,40] have been devoted to the phase behavior of polyelectrolyte solutions. Michaeli, Overbeek, and Voorn [33,34] showed that phase separation may arise in solutions of polyelectrolytes due to electrostatic correlations using the generalized Flory–Huggins theory and the Debye–Hückel theory. Moreover, Jiang et al. [41,42,43] developed a thermodynamic theory based on the simplified charged hard-sphere chain model to study the phase equilibria of polyelectrolyte solutions. Such a theoretical framework was further developed by Zhang et al. [44], who presented what is referred to as a liquid-state (LS) theory to predict the phase behavior of fully-charged polymer solutions in both salt-free conditions and with added salt. This LS theory, which accounts for hard-core excluded volume repulsion by the Boublík–Mansoori–Carnahan–Starling–Leland (BMCSL) [45,46] equation of state, electrostatic correlation by mean-spherical approximation (MSA) [47,48,49], and chain connectivity by Wertheim’s first order thermodynamic perturbation theory (TPT1) [50,51,52,53,54], has shown remarkable success in predicting the phase behavior of polyelectrolyte solutions [44,55,56]. Such an LS theory has been applied to study the phase behaviors of concentration-asymmetric mixtures of polycation and polyanion solutions, and has revealed a wealth of interesting and complex phase separation scenarios [57]. Classical density functional theories (cDFT) for charged polymers have been developed based on a similar framework, and have found wide application in many polyelectrolyte systems [58,59,60,61,62,63,64,65]. A complete theoretical understanding of the solution phase behaviors of charged polymers, however, remains challenging, both because of the multi-component nature of the system (which, in the simplest case of a salt-free solution of fully charged polymers, consists of solvent, counterions, and charged polymers) and because of the delicate interplay among various factors, including the translational entropy of each component, excluded volume interactions, chain connectivity, and more importantly, the long-range electrostatic interactions. Considering the complexity of the systems, most polyelectrolyte models in theoretical studies focus on electrostatic interactions and hard-sphere type excluded volume interactions, and ignore the effects of local short-range interactions [66,67,68,69] such as hydrogen bonding and dipolar interactions [70,71,72,73,74], hydrophobic interactions [75,76,77,78,79,80], specific ion binding interactions [81,82,83,84,85,86,87,88,89,90], and couplings among them.

In a recent work [91], the LS theory of Zhang et al. [44] was applied to study the phase behavior of partially charged ionic polymers in both the salt-free case and with salt added 2:1. Previous studies have shown that there can be an additional short-range attraction, often referred to as the “calcium-binding” interaction [92,93,94,95,96,97], between calcium ions and the negatively-charged carboxylate groups of polycarboxylate-based superplasticizers (PCEs) [13]. Such a calcium-binding interaction and how its strength affects the phase behavior were investigated in our earlier work by introducing a modified square well potential for the ${\mathrm{Ca}}^{2+}$ and $R-{\mathrm{CO}}_{2}^{-}$ pairs, which was incorporated into the LS theory by an extra Helmholtz free energy from the perturbed-chain statistical associating fluid theory (PC-SAFT) [98,99]. We found that increasing the calcium-binding strength expands the phase-separated region and increases the critical extra salt concentration, and leads to a wider phase-separated region for salting-out and salting-in phenomena. The structural parameters of PCEs affect phase behavior as well. Increasing the length of the neutral side chains shrinks the phase-separated region, while increasing the acid-to-ether ratio expands the phase-separated region. A combination of PC-SAFT and cDFT for charged polymers has found applications in studies of the thermodynamically responsive properties of a grafted polyanion layer on a planar surface [63] and the effects of polyelectrolyte surface coating on the energy storage performance of supercapacitors [64].

In the present work, we consider a linear polymer consisting of two types of monomers, A (charged) and B (neutral), in a polar solvent. The counterions released are referred to as type C. In a recent work by Qiu et al. [91], we considered only dispersion interactions between A and C, which are often referred to as specific ion binding interactions. In the present work, dispersion interactions between A and A and between B and B are considered. In addition to reporting the phase diagrams of such a system and how they are affected by the various parameters, this work presents a graphical user interface application (GUI App) that allows users to calculate such phase diagrams by inserting the values of the model parameters. The rest of this paper is organized as follows. Section 2 presents the system of interest, the model, the theoretical framework and its details, and the numerical methods. Section 3 presents the key results of this work, and Section 4 concludes the paper. In the Supporting Materials, the Matlab (version R2022a) codes that were used to produce the results and the GUI App presented in the paper are presented.

## 2. Model and Methods

### 2.1. Polymer and Solution Models

**Figure 1:**
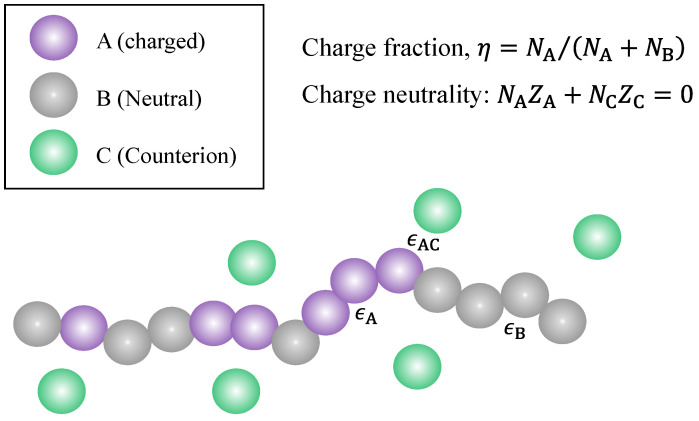
Illustration of a partially charged polymer with total length *N*, a sum of the charged ($N_{A}$) segments (type A) and neutral ($N_{B}$) segments (type B) and its counterions (type C). There are short-range interactions between A and C, between A and A, and between B and B.

Figure 1 presents the model system considered in this work. It is a coarse-grained model where chain segments are described by spherical beads, a common approach in the modeling of polymers [100,101,102]. We consider a linear copolymer made up of two types of segments, A and B, where A is charged with valence $Z_{A}$ (i.e., every A segment carries an electric charge of $Z_{A}$ in units of the elementary charge) and B is uncharged. Note that if the ionic groups are from weak acids or bases that are only partially ionized [103,104], then A and B may have nearly the same chemical structure except that one is ionized and the other one is not. Let $N_{A}$ and $N_{B}$ be the number of A segments and the number of B segments in the polymer, respectively; then, the polymer has $N_{T}\equiv N_{A}+N_{B}$ segments in total and has a charge fraction

(1) $$\eta =\frac{N_{A}}{N_{T}}=\frac{N_{A}}{N_{A}+N_{B}}$$

Furthermore, the condition of charge neutrality for a single chain (i.e., polymer) provides

(2) $$N_{A}Z_{A}+N_{C}Z_{C}=0$$

where $N_{C}$ is the total number of counterions associated with a single chain and $Z_{C}$ is the valence of a single counterion.

In connection with the real world, the model system considered here may be relevant to weak polyelectrolytes [9,10] such as Poly(acrylic acid) (PAA) brushes and poly(methacrylic acid) (PMAA), partially hydrolyzed polyacrylamide (HPAM) [105,106,107], hydrophobically modified polyelectrolytes [77,108,109] or an approximation, partially hydrolyzed hydrophobically modified polyacrylamide [110], or hydrophobically modified PCEs [111,112]. Such polymers have important applications as absorbents, polymeric dispersants, polymer flooding for enhanced oil recovery, wastewater treatment, and concrete admixtures.

**Figure 2:**
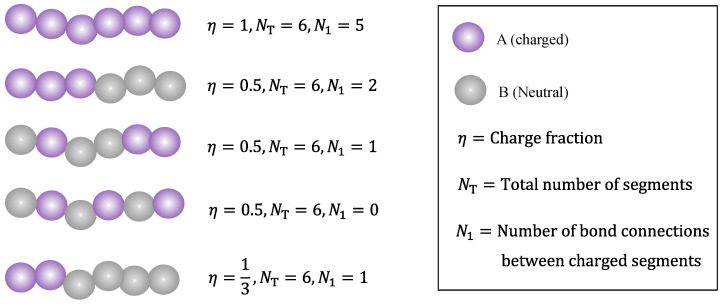
Examples illustrating how the charge fraction η and sequence distribution (from top to bottom: fully charged, block copolymer, random copolymer, alternating copolymer, and block copolymer) affect the relationship between the number of bond connections among charged beads and the total number of chain segments.

It is well known in polymer science that copolymers can be random (statistical), blocks, alternating, etc., in terms of sequence distribution. As is shown in the next section, while the present work does not provide a complete description of how the monomer sequence distribution affects its phase behavior, there is indeed one parameter in the model that depends on the sequence distribution, i.e., the number of bond connections among charged beads, denoted by $N_{1}$. For a fully charged (charge fraction, η=1) linear polyelectrolyte chain, we have $N_{1}=N_{T}-1$. However, depending on the charge fraction and monomer sequence distribution, $N_{1}$ can be substantially smaller than $N_{T}$. Figure 2 demonstrates schematically how the polymer charge fraction and monomer sequence distribution affect the relationship between the number of bond connections among charged beads and the total number of chain segments.

**Figure 3:**
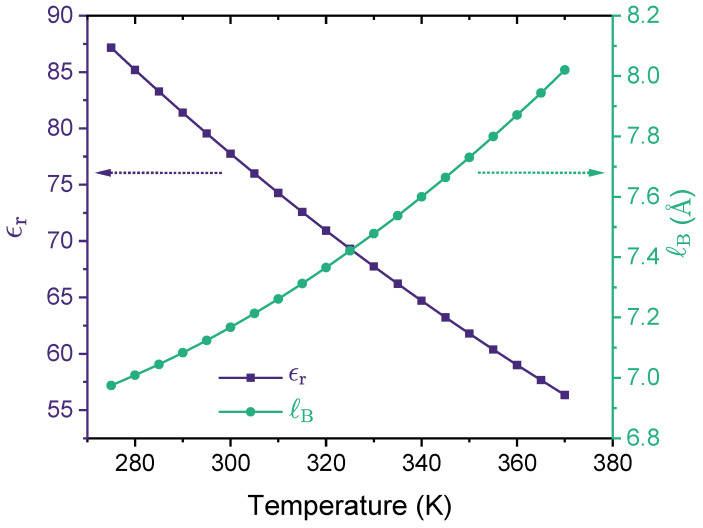
Relative dielectric constant $\epsilon_{r}$ (left y-axis) and the corresponding Bjerrum length $\ell_{B}$ (right y-axis) are shown as a function of temperature for liquid water at 1 atm. In preparing this figure, we used Malmberg and Maryott’s empirical model [114] of the dielectric constant of water, $\epsilon_{r}\left(T\right)=87.740-0.40008T+9.398\times 10^{-4}T^{2}-1.410\times 10^{-6}T^{3}$, where *T* is the temperature value in degrees Celsius (°C).

The solvent is treated as a dielectric continuum with a dielectric constant $\epsilon_{r}$. The electrostatic interaction between any two charged beads with valences $Z_{i}$ and $Z_{j}$ and separated by a distance $r_{ij}$ is described by a superposition of the Coulomb potential and the hard-sphere potential:

(3) $$U_{\mathrm{Coul}}\left(r_{ij}\right)=\left\{\begin{array}{cc} \beta^{-1}Z_{i}Z_{j}\ell_{B}/r_{ij} & \;r_{ij}>\sigma_{ij}\\ \infty & \;r_{ij}\leq \sigma_{ij} \end{array}\right.$$

where $\beta^{-1}\equiv k_{B}T$ is the thermal energy scale, $k_{B}$ is the Boltzmann constant, *T* is the absolute temperature in degrees Kelvin, $\sigma_{ij}$ refers to the effective radii between the two beads (*i* and *j*) of diameter $\sigma_{i}$ and $\sigma_{j}$, respectively. By the Lorentz rule, $\sigma_{ij}=\left(\sigma_{i}+\sigma_{j}\right)/2$, while $\ell_{B}$ is the Bjerrum length, which is the separation at which the electrostatic interaction between two elementary charges is comparable in magnitude to the thermal energy scale $k_{B}T$. In standard units, $\ell_{B}$ is provided by

(4) $$\ell_{B}=\frac{e^{2}}{4\pi \epsilon_{0}\epsilon_{r}k_{B}T}$$

where *e* is the elementary charge, $\epsilon_{r}$ is the relative dielectric constant of the medium, and $\epsilon_{0}$ is the vacuum permittivity. For water at room temperature (T≈293K), $\epsilon_{r}\approx 80$, meaning that $\ell_{B}\approx 7.1\;Å$. It appears from Equation (4) that $\ell_{B}$ is proportional to $T^{-1}$ such that the higher the temperature, the lower the Bjerrum length. However, this notion is only correct when $\epsilon_{r}$ is treated as a constant, i.e., independent of temperature. For real liquids, the relative dielectric constant depends on the temperature, i.e., $\epsilon_{r}=\epsilon_{r}\left(T\right)$. In the case of liquid water at 1 atm, as shown by Figure 3, $\epsilon_{r}$ decreases with the increase of temperature [56,113,114], which leads to an increase of $\ell_{B}$ as the temperature increases. In the temperature range for liquid water at 1 atm, $\ell_{B}$ falls in the interval of [7.0Å,8.0Å]. As recently reported by Ylitalo and coworkers [56], accounting for the temperature dependence of the dielectric constant of water is essential when modeling a lower critical solution temperature (LCST) because it results in a Bjerrum length that increases (rather than decreases) with temperature, leading to stronger electrostatic correlations that drive phase separation at higher temperatures.

Pairwise Dispersion interactions between A and A, between B and B, and between A and C are considered in the present work. Their interaction strengths are described by three energy (in the unit of $k_{B}T$) parameters: $\epsilon_{A}$ for the dispersion interaction between A and A, $\epsilon_{B}$ for that between B and B, and $\epsilon_{AC}$ for the dispersion interaction between A and C, as shown schematically in Figure 1. The pair potential for the dispersion interactions between pair species is provided by a modified square well potential, which was suggested by Chen and Kreglewski [98] and used in the PC-SAFT equation of state developed by Gross and Sadowski [99]. This pair potential between two beads separated by a distance $r_{ij}$ is provided by

(5) $$\beta U_{\mathrm{disp}}\left(r_{ij}\right)=\left\{\begin{array}{cc} \infty & r_{ij}<\lambda_{1}\sigma_{ij}\\ 3\epsilon^{*} & \lambda_{1}\sigma_{ij}\leq r_{ij}<\sigma_{ij}\\ -\epsilon^{*} & \sigma_{ij}<r_{ij}<\lambda_{2}\sigma_{ij}\\ 0 & r_{ij}\geq \lambda_{2}\sigma_{ij} \end{array}\right.$$

where $\sigma_{ij}=\left(\sigma_{i}+\sigma_{j}\right)/2$, $\lambda_{1}=0.88$ and $\lambda_{2}=1.5$ are two parameters from the PC-SAFT model, and $\epsilon^{*}$ (dimensionless), which can be $\epsilon_{A}$, $\epsilon_{B}$, or $\epsilon_{AC}$ in this work, is the depth of the potential well relative to the thermal energy scale. In this work, we limited ourselves to considering pairwise dispersion interactions between A and A, between B and B, and between A and C, which represent specific interactions between the charged segments of the polymer, between the neutral segments of the polymer, and between the charged polymer segments and their counterions, respectively. In principle, all other dispersion interactions, which are assumed to be pairwise additive, could be introduced into the model by extending the source codes provided.

### 2.2. Theoretical Formulation

Table 1 presents a summary of the different species in the systems examined in this work. For the more general case of a polymer solution with added salt, after ionization in a polar solvent (described as a dielectric continuum), there are four types of beads in the solution: Type A for the charged segments of the polymer, Type B for the neutral segments of the polymer, Type C for the counterions of the charged segments of the polymer and salt co-ions, and type D for the co-ions from the added salt (assumed to be C+D). For the simplicity of writing, we denote the whole polymer by “p” (which consists of type A and type B segments), and the polymer segmental density by $\rho_{p}=\rho_{A}+\rho_{B}$. In this study, we mainly focus on the salt-free polymer solution case where $\rho_{D}=0$.

The theoretical framework and LS theory used in this study to predict the phase behavior of ion-containing polymers in polar solvents are similar to those reported in a recent work by Qiu et al. [91]. The LS theory was adapted from that developed by Zhang et al. [44,55,57] for the phase behavior and salt partitioning of polyelectrolyte solutions, and dispersion interactions were introduced to the LS theory by an additional free energy term from the PC-SAFT equation of state, following the recent work by Xu and coworkers [63,115]. The system Helmholtz free energy density *f* can generally be written as the sum of an ideal contribution ($f^{\mathrm{id}}$) and an excess contribution ($f^{\mathrm{ex}}$) [44]:

(6) $$f=f^{\mathrm{id}}+f^{\mathrm{ex}}$$

The ideal part $f^{\mathrm{id}}$ describes the translational degrees of freedom, and is known exactly [44]:

(7) $$\beta f^{\mathrm{id}}=\frac{\rho_{p}}{N_{T}}\left[\ln\left(\frac{\rho_{p}}{N_{T}}\Lambda_{p}^{3}\right)-1\right]+\sum_{i=C,D}\rho_{i}\left[\ln\left(\rho_{i}\Lambda_{i}^{3}\right)-1\right]$$

where $\rho_{p}=\rho_{A}+\rho_{B}$, $N_{T}=N_{A}+N_{B}$, and $\Lambda_{p}$ and $\Lambda_{i}$ are length scales arising from integrals over the momentum degrees of freedom; while they are kept in Equation (7) for dimensional consistency, they have no consequences with respect to the phase behavior. In this theoretical framework, $f^{\mathrm{id}}$ describes only the translational entropy, and does not distinguish the difference between neutral and charged segments of the polymer. Hence, the total number of beads of a polymer, $N_{T}$, is used in Equation (7).

The excess Helmholtz free energy density is provided by [91]:

(8) $$f^{\mathrm{ex}}=f_{\mathrm{hs}}^{\mathrm{ex}}+f_{\mathrm{el}}^{\mathrm{ex}}+f_{\mathrm{ch}}^{\mathrm{ex}}+f_{\mathrm{disp}}^{\mathrm{ex}}$$

The first term on the right side of Equation (8), $f_{\mathrm{hs}}^{\mathrm{ex}}$, represents the contribution from the hard-core excluded volume interactions (see Equation (3)) of an ensemble of “disconnected” (assumed) hard spheres. This contribution is described by the BMCSL excess free energy density [45,46]:

(9) $$\beta f_{\mathrm{hs}}^{\mathrm{ex}}=-\xi_{0}\ln\left(1-\xi_{3}\right)+\frac{\xi_{1}\xi_{2}}{1-\xi_{3}}+\frac{\xi_{2}^{3}}{3}\left[\frac{\ln(1-\xi_{3})}{12\pi \xi_{3}^{2}}+\frac{1}{12\pi \xi_{3}{\left(1-\xi_{3}\right)}^{2}}\right]$$

with $\xi_{0}\equiv \sum_{i}\xi_{0i}=\sum_{i}\rho_{i}$, $\xi_{1}\equiv \sum_{i}\xi_{1i}=\left(1/2\right)\left(\sum_{i}\rho_{i}\sigma_{i}\right)$, $\xi_{2}\equiv \sum_{i}\xi_{2i}=\pi \sum_{i}\rho_{i}\sigma_{i}^{2}$, and $\xi_{3}\equiv \sum_{i}\xi_{3i}=\left(\pi /6\right)\sum_{i}\rho_{i}\sigma_{i}^{3}$, where the sum over *i* spans all species, A, B, C, and D (see Table 1).

The electrostatic correlation of the “disconnected” (assumed) charged hard spheres can be accounted for using the Ornstein–Zernike equation with MSA [116], and the corresponding excess free energy density, $f_{\mathrm{el}}^{\mathrm{ex}}$, is obtained as [44]

(10) $$\beta f_{\mathrm{el}}^{\mathrm{ex}}=-\ell_{B}\sum_{i}\frac{\xi_{0i}Z_{i}}{1+\sigma_{i}\Gamma }\left[Z_{i}\Gamma +\frac{\pi \sigma_{i}}{2}\frac{P_{n}}{1-\xi_{3}}\right]+\frac{1}{3\pi }\Gamma^{3}$$

where the sum over *i* spans all species, A, B, C, and D (see Table 1). The screening parameter Γ and the size asymmetric factor $P_{n}$ are obtained from the following set of equations [44]:

(11) $$\begin{array}{c} \left\{\begin{array}{cc} & \Gamma^{2}=\pi \ell_{B}\sum_{i}\frac{\xi_{0i}}{{\left(1+\sigma_{i}\Gamma \right)}^{2}}{\left[Z_{i}-\frac{\pi \sigma_{i}^{2}}{2}\frac{P_{n}}{1-\xi_{3}}\right]}^{2}\\ & P_{n}=\left.\sum_{i}\frac{2\xi_{1i}Z_{i}}{1+\sigma_{i}\Gamma }/\left[1+\frac{3}{1-\xi_{3}}\sum_{i}\frac{\xi_{3i}}{1+\sigma_{i}\Gamma }\right]\right. \end{array}\right. \end{array}$$

The third term on the right side of Equation (8), $f_{\mathrm{ch}}^{\mathrm{ex}}$, represents a correction due to chain connectivity. For the systems considered in this study, there are two types of connections, namely, connections between charged beads and the remaining connections. Let $N_{1}$ and $N_{2}$ be the number of connections among charged beads and the number of remaining connections, respectively. The excess Helmholtz free energy density due to chain connectivity may be considered as a sum of two contributions, one for the connectivity among charged beads and the other from the remaining connections. For the former, we use the same expression (rewritten in a slightly different form) as that used by Zhang et al. [44,55,57], while for the latter we use the result from TPT1, originally proposed by Wertheim for neutral hard-sphere chain systems [50,54]. In brief, $f_{\mathrm{ch}}^{\mathrm{ex}}$ is obtained as follows:

(12) $$\begin{array}{ccc} \beta f_{\mathrm{ch}}^{\mathrm{ex}} & = & -\frac{1}{N_{T}}\xi_{0p}\left\{N_{1}\ln\left[\frac{1}{1-\xi_{3}}+\frac{\xi_{2}\sigma_{p}}{4{\left(1-\xi_{3}\right)}^{2}}\right]\exp\left(-\frac{a_{1}^{2}\Gamma^{2}}{4\pi^{2}\sigma_{p}\ell_{B}}+\frac{\ell_{B}Z_{A}^{2}}{\sigma_{p}}\right)\right.\\ \; & \; & +\left.\left(N_{T}-1-N_{1}\right)\ln\left[\frac{1}{1-\xi_{3}}+\frac{\xi_{2}\sigma_{p}}{4{\left(1-\xi_{3}\right)}^{2}}\right]\right\} \end{array}$$

where $N_{1}$ is the number of bond connections between two charged segments (type A), $N_{T}-1-N_{1}$ is the remaining number of bond connections, $\xi_{0p}=\rho_{p}=\rho_{A}+\rho_{B}$ according to the definition of $\xi_{0i}$, $\sigma_{p}=\sigma_{A}=\sigma_{B}$, and

(13) $$a_{1}=\frac{2\pi \ell_{B}\left[Z_{A}-\pi P_{n}\sigma_{p}^{2}/\left(1-\xi_{3}\right)\right]}{\left(1+\sigma_{p}\Gamma \right)\Gamma }$$

In Equation (12), $N_{1}$ is the number of bond connections among charged beads, as is illustrated in Figure 2 for partially charged polymers, while $N_{1}$ depends on the monomer sequence distribution and is used in the present theory to describe the influence of sequence distribution on the polymer phase behavior.

The last term on the right side of Equation (8), $f_{\mathrm{disp}}$, represents a contribution from dispersion interactions. In this work, we consider three types of short-range interactions: (i) between counterions C and charged segments A with strength parameter $\epsilon_{AC}$, (ii) between two charged segments, A and A, with strength parameter $\epsilon_{A}$, and (iii) between two neutral segments, B and B, with strength parameter $\epsilon_{B}$. From the PC-SAFT model [99,115], we have

(14) $$f_{\mathrm{disp}}=f_{\mathrm{disp}}^{\mathrm{AC}}+f_{\mathrm{disp}}^{\mathrm{AA}}+f_{\mathrm{disp}}^{\mathrm{BB}}$$

where

(15) $$\left\{\begin{array}{c} \beta f_{\mathrm{disp}}^{\mathrm{AC}}=-2\pi \rho_{A}\rho_{C}\left[2J_{1}\epsilon_{AC}+\bar{N}M^{-1}J_{2}{\epsilon_{AC}}^{2}\right]\sigma_{A,C}^{3}\\ \beta f_{\mathrm{disp}}^{\mathrm{AA}}=-2\pi \rho_{A}\rho_{A}\left[2J_{1}\epsilon_{A}+\bar{N}M^{-1}J_{2}{\epsilon_{A}}^{2}\right]\sigma_{A}^{3}\\ \beta f_{\mathrm{disp}}^{\mathrm{BB}}=-2\pi \rho_{B}\rho_{B}\left[2J_{1}\epsilon_{B}+\bar{N}M^{-1}J_{2}{\epsilon_{B}}^{2}\right]\sigma_{B}^{3} \end{array}\right.$$

with

(16) $$M=1+\bar{N}\frac{8\xi_{3}-2\xi_{3}^{2}}{{\left(1-\xi_{3}\right)}^{2}}+\left(1-\bar{N}\right)\frac{20\xi_{3}-27\xi_{3}^{2}+12\xi_{3}^{3}-2\xi_{3}^{4}}{{\left(1-\xi_{3}\right)}^{2}{\left(2-\xi_{3}\right)}^{2}}$$

Here, $\sigma_{A,C}=\left(\sigma_{A}+\sigma_{C}\right)/2$ by the Lorentz rule; $\xi_{3}\equiv \sum_{i}\xi_{3i}=\left(\pi /6\right)\sum_{i}\rho_{i}\sigma_{i}^{3}$, as defined underneath Equation (9), although here the sum over *i* runs only over the associating species, A, B, and C; and $\bar{N}$ is a weight-average number of segments of the associating species:

(17) $$\bar{N}=\frac{N_{T}\rho_{p}+\rho_{C}}{\rho_{p}+\rho_{C}}$$

In Equation (15), $J_{1}$ and $J_{2}$ refer to contributions from integrals of the radial distribution function, and are obtained as six-order polynomials [99] in the form of

$$J_{k}=\sum_{i=0}^{6}a_{i}^{\left(k\right)}\left(\bar{N}\right)\xi_{3}^{i}\;,\;k=1,\;2$$

with the coefficients

$$a_{i}^{\left(k\right)}\left(\bar{N}\right)=a_{i0}^{\left(k\right)}+\frac{\bar{N}-1}{\bar{N}}a_{i1}^{\left(k\right)}+\frac{\bar{N}-1}{\bar{N}}\frac{\bar{N}-2}{\bar{N}}a_{i2}^{\left(k\right)}$$

where $a_{ij}^{\left(k\right)},,k=1,\;2$, are the universal model constants fixed by Gross and Sadowski [99] in their development of PC-SAFT.

More recently, Zhang et al. [117] have presented a newer version of LS theory which can capture the electrostatic correlations due to chain connectivity more accurately for partially charged polymers. Although the present work is based on their earlier version of the LS theory [44], we do not expect qualitative differences in the predicted phase diagrams. A comparative study can be made in the future to quantify the differences between those two versions of the LS theory by incorporating the new theory into the Matlab codes presented in this work.

### 2.3. Construction of Phase Diagram

Phase equilibrium between the polymer-poor phase (denoted as phase I) and the polymer-rich phase (denoted as phase II) is determined by the equality of the electrochemical potential for all components (see Table 1) and the equality of the osmotic pressure [44]. Charge neutrality is enforced by introducing a Lagrange multiplier Ψ in the grand free energy minimization, similar to the approach conducted in earlier studies [44,118,119]. We have

(18) $$F=f+e\Psi \sum_{k}\rho_{k}Z_{k}\;k=A,\;B,\;C,\;D$$

where the Lagrange multiplier Ψ has the interpretation of an electrostatic potential. The equality of the electrochemical potential for all components leads to

(19) $$\frac{\partial F^{I}}{\partial \rho_{k}^{I}}=\frac{\partial F^{\mathrm{II}}}{\partial \rho_{k}^{\mathrm{II}}}$$

The superscripts I and II are introduced here to specify the polymer-poor phase and the polymer-rich phase, respectively. Recall that the chemical potential of each species, $\mu_{i}$, is obtained from the overall Helmholtz free energy density and is provided by [44]

(20) $$\mu_{k}=\frac{\partial f}{\partial \rho_{k}}\;,\;k\equiv p,\;C,\;D$$

The equality of the electrochemical potential for all components, provided by Equation (19), thus leads to

(21) $$\begin{array}{ccc} \mu_{j}^{I}+eZ_{j}\Psi^{I} & = & \mu_{j}^{\mathrm{II}}+eZ_{j}\Psi^{\mathrm{II}}\;,\;j=C,\;D \end{array}$$

(22) $$\begin{array}{ccc} \mu_{p}^{I}+eZ_{A}\eta \Psi^{I} & = & \mu_{p}^{\mathrm{II}}+eZ_{A}\eta \Psi^{\mathrm{II}} \end{array}$$

Equations (21) and (22) denote the equality of the electrochemical potential for all charged species (C, D, and p), while for the polymer species p the valence is reduced to $\eta Z_{A}$ because of its partially charged characteristics. While there are two Lagrange multipliers, $\Psi^{I}$ and $\Psi^{\mathrm{II}}$, for the polymer-poor and the polymer rich phases, respectively, only their difference is meaningful, which can be understood as a Galvani potential ($\Psi_{G}$) [44], defined as $\Psi_{G}\equiv \Psi^{\mathrm{II}}-\Psi^{I}$. Using the Galvani potential, Equations (21) and (22) may be rewritten as

(23) $$\begin{array}{ccc} \mu_{j}^{I}-\mu_{j}^{\mathrm{II}} & = & eZ_{j}\Psi_{G}\;,\;j=C,\;D \end{array}$$

(24) $$\begin{array}{ccc} \mu_{p}^{I}-\mu_{p}^{\mathrm{II}} & = & eZ_{A}\eta \Psi_{G} \end{array}$$

The equality of the osmotic pressure (provided by P=∑ρμ−f) [44] leads to

(25) $$\sum_{k}\mu_{k}^{I}\rho_{k}^{I}-f^{I}=\sum_{k}\mu_{k}^{\mathrm{II}}\rho_{k}^{\mathrm{II}}-f^{\mathrm{II}}\;,\;k=p,\;C,\;D$$

The charge neutrality constraint is provided by

(26) $$\frac{\partial F^{I}}{\partial \Psi^{I}}=\frac{\partial F^{\mathrm{II}}}{\partial \Psi^{\mathrm{II}}}=0$$

which may be rewritten as

(27) $$\sum_{k}\rho_{k}^{I}Z_{k}=\sum_{k}\rho_{k}^{\mathrm{II}}Z_{k}=0\;k=A,\;B,\;C,\;D$$

Equation (25) denotes the equality of the osmotic pressure of phase I and that of phase II, and Equation (27) denotes the charge neutrality constraint in each of the phases. To construct the phase diagram, Equations (23)–(25) and (27) are solved to obtain the density of the species in each of the phases and the Galvani potential.

Let *F* be the number of degrees of freedom *C* be the number of components and let Ph be the number of phases. According to the Gibbs phase rule, F=C−Ph+2. For the salt-free case (polymer with counterions only), because of charge neutrality there is only one independent component, i.e., C=1; for such a system at two-phase coexistence (Ph=2), there should only be one degree of freedom, i.e., F=1, and its phase diagrams can be drawn by specifying a $\ell_{B}$ value. Applying the same analysis to solutions with salt, we have F=2, and the resulting phase diagram is a curved surface in 3D, which is not easy to illustrate or understand. In this study, such phase diagrams are constructed by varying the osmotic pressure (Equation (25)) at a fixed $\ell_{B}$ value.

The detailed numerical procedure was explained in an earlier work [91]. The globally convergent Newton method [120] is employed to minimize the errors (tolerance $<10^{-12}$) when solving the system of nonlinear equations. We have assembled our Matlab (version R2022a) codes for the salt-free case into a GUI App (introduced in Section sec:Results), and the codes are provided in the Supplementary Materials.

In this work, we consider all beads (monomer segments, counterions, salt ions) to have the same diameter of 4.0Å, following the work of Zhang et al. [44,57]. For simplicity of notation, we use σ to denote the bead diameter, i.e., $\sigma =\sigma_{A}=\sigma_{B}=\sigma_{C}=\sigma_{D}=4.0\;Å$. The segmental number densities $\rho_{i}$ are expressed in units of $\sigma^{-3}$. In other words, $\rho_{i}\sigma^{3}$ is dimensionless. With σ=4.0Å, a polymer segmental number density of $\rho_{p}\sigma^{3}=0.01$ corresponds to a polymer concentration of

(28) $$\frac{\rho_{p}}{N_{T}}=\frac{0.01\sigma^{-3}}{N_{T}}=\frac{0.01\times {\left(4.0\;Å\times 10^{-9}\;\mathrm{dm}/Å\right)}^{-3}}{N_{T}}\frac{1}{N_{A}}\approx 0.26N_{T}^{-1}\;\mathrm{mol}/L$$

where $N_{T}$ is the total number of segments of the polymer. Note that it is possible to consider beads of different sizes, as was done in our recent work [91], and to consider bead diameter as a function of temperature, as described in the PC-SAFT literature [99] and a recent work by Xu et al. [63]. However, because the theoretical formulation presented in this section is already complicated, in pursue of a minimalist model such detailed factors are not included in this study; we do not expect qualitative differences from the results presented in this work when those factors are included.

## 3. Results and Discussions

### 3.1. GUI App for the Salt-Free Case and Selected Sample Results

**Figure 4:**
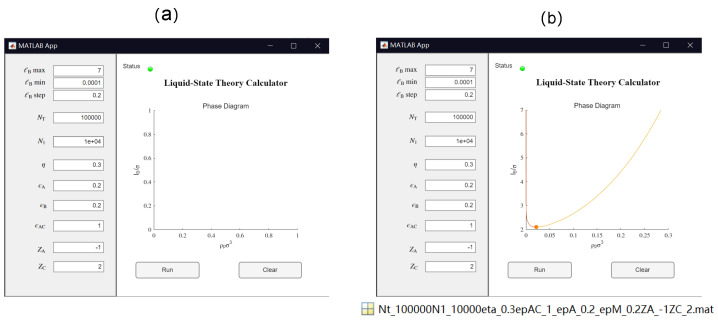
Illustration of the GUI App of a Liquid State Theory Calculator based on MATLAB (version R2022a): (**a**) after setting values for the parameters but before clicking “Run” and (**b**) the resulting binodal curve and critical point (shown by the filled circle). Here, the polymer solution is in a single phase below the binodal curve and is separated into two phases above the binodal curve. The App generates a Matlab “.mat” file containing the numerical results labeled by the parameters for further analysis.

Inspired by the work of Thompson et al., efforts have been made in this study to make the theoretical model more transparent, reproducible, usable by others, and extensible (TRUE) [121]. As a result, in this work we have developed a GUI App based on MATLAB (version R2022a), which allows for convenient calculation of the binodal curve for the salt-free case. All the source codes are supplied in the Supplementary Materials. Table 2 summarizes the list of parameters in the model and in the GUI App. After the values for these parameters are set, the App calculates the corresponding phase diagram for the salt-free case by clicking the “Run” button. The calculated result are shown in the GUI. Users can use the App to easily compare the results from different sets of parameters. The App can generate a Matlab “.mat” file of the numerical results labeled by the parameters for further analysis (see Figure 4). The source codes for the App and the traditional Matlab Run file are provided for further extensions of the code.

The resulting binodal curve, which separates one phase and two phases systems, is shown in the form of $\ell_{B}/\sigma$ vs. $\rho_{p}\sigma^{3}$ (see Figure 4b). As $\ell_{B}$ depends on temperature (see Equation (4) and Figure 3), such an $\ell_{B}/\sigma$ vs. $\rho_{p}\sigma^{3}$ binodal curve can be easily mapped to the transitional form of a temperature vs. composition binodal curve. On a $\ell_{B}/\sigma$ vs. $\rho_{p}\sigma^{3}$ graph, the polymer solution is in two-phase coexistence above the binodal and is a single phase below the binodal. The critical point is depicted on the binodal curve as a filled circle (see Figure 4).

**Figure 5:**
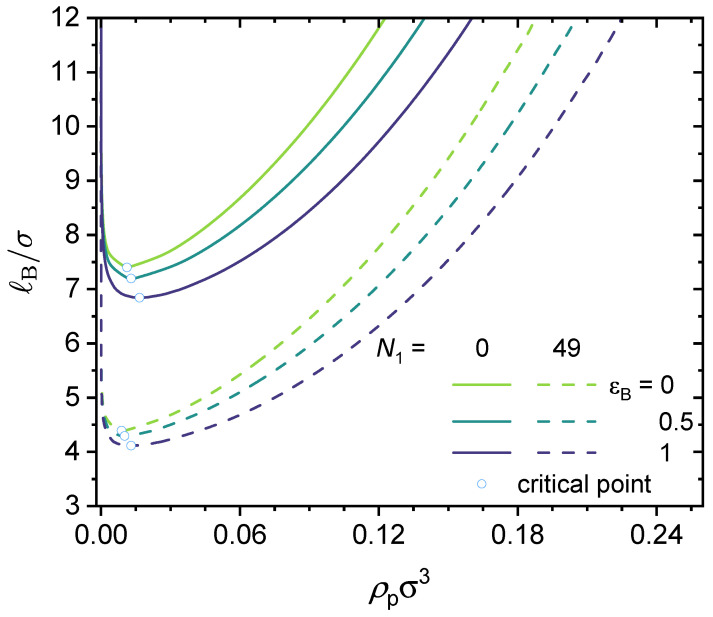
Predicted binodal curves from the GUI App presented in this work. The values for the following parameters were fixed: $N_{T}=100$, η=0.5, $\epsilon_{AC}=\epsilon_{A}=0$, $Z_{A}=-1$, $Z_{C}=+1$. Values for $N_{1}$ and $\epsilon_{B}$ were varied, as shown in the figure. Critical points are shown as open circles.

**Figure 6:**
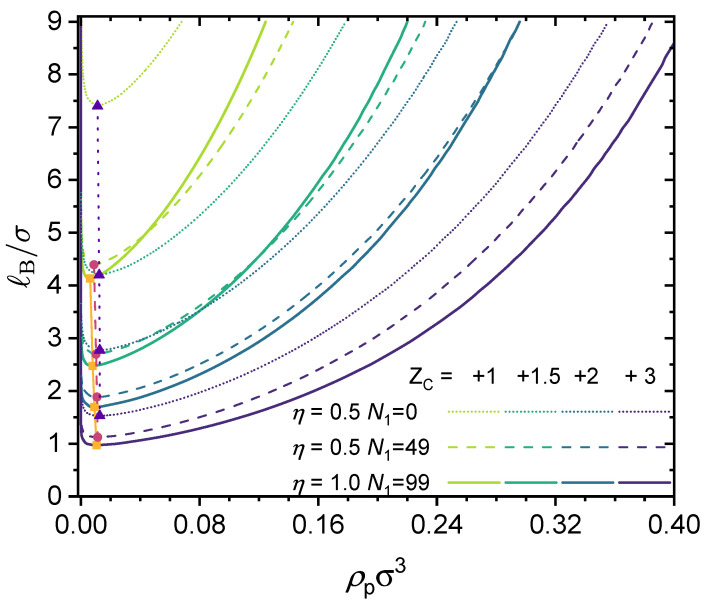
Predicted binodal curves from the GUI App presented in this work. The values for the following parameters were fixed: $N_{T}=100$, $\epsilon_{AC}=\epsilon_{A}=\epsilon_{B}=0$, $Z_{A}=-1$. Values for η, $N_{1}$ and $Z_{C}$ were varied, as shown in the figure. Critical points are depicted by filled symbols.

In this section, we present results from two sample problems to demonstrate how such results can be obtained easily using the GUI App developed in this work. Note that the theoretical model presented in Section 2 is a deterministic model and there is no randomness in the results other than truncation errors. The same set of parameters should always generate the same results, and therefore readers may compare their results with the results shown in this section to check whether the App was run properly. In both cases (see Figure 5 and Figure 6), the total number of segments of the polymer was fixed at $N_{T}=100$. Note that the relationship between the charge fraction η and the number of bond connections between charged segments $N_{1}$ is nontrivial. For block copolymers (see Figure 2), we have $N_{1}=\eta N_{T}-1$, which is the upper bound for the $N_{1}$ parameter when η and $N_{T}$ are fixed. For other types of monomer sequence distributions apart from a block copolymer, the corresponding $N_{1}$ values would be smaller than $\eta N_{T}-1$, leaving the question of what the lower bound for $N_{1}$ is. As shown in Figure 2, compared to other types of monomer sequence distributions, the alternating monomer sequence distribution leads to a lower $N_{1}$ value; if η≤0.5, then the lower bound for $N_{1}$ would be 0, i.e., $N_{1}\in \left[0,\eta N_{T}-1\right]$. However, if the charge fraction is notably larger than 0.5, then the lower bound for $N_{1}$ would be notably larger than 0. A more careful analysis of the influence of charge fraction and monomer sequence distribution on the range of $N_{1}$ shows that the lower bound for $N_{1}$ is

(29) $$N_{1,\min}=\max\left[0,N_{T}\left(2\eta -1\right)-1\right]$$

Therefore, we have $N_{1}\in \left[N_{1,\min},\eta N_{T}-1\right]$. For example, considering a polymer chain with 100 beads and a charge fraction of η=0.7, the acceptable range of $N_{1}$ is from 100×(2×0.7−1)−1=39 to 100×0.7−1=69. For the results shown in Figure 5, we have $N_{T}=100$ and η=0.5. Therefore, the acceptable range of $N_{1}$ is from 0 to 49, with the lower bound corresponding to an alternating copolymer and the upper bound corresponding to a block copolymer. Here, we only consider short-range dispersion interactions between the neutral segments (type B) in the polymer, i.e., we set $\epsilon_{A}=\epsilon_{AC}=0$ and vary $\epsilon_{B}$ from 0 (no interaction) to 1 (short-range dispersion interaction on the order of the thermal energy).

As shown in Figure 5, increasing the $N_{1}$ value expands the phase-separated region. In other words, increasing the $N_{1}$ value promotes phase separation. That is to say, for the same charge fraction, a block copolymer would have a wider phase-separated region than an alternating copolymer. Similarly, for a given $N_{1}$ value, an increase in $\epsilon_{B}$, which is the strength of the dispersion interactions between the neutral segments, promotes phase separation. We now turn to the underlying physics. As shown in the theoretical formulation, the translational entropy term promotes mixing and works against phase separation; however, the electrostatic correlation effects, chain connectivity, and short-range dispersion interactions all weaken the entropy term, and thus promote phase separation. This notion agrees with the results shown in Figure 5.

The results shown in Figure 5 are for monovalent counterions, i.e., $Z_{C}=+1$. In Figure 6, the valency of the counterions is varied, and results are shown for three different polymers: (i) an alternating copolymer with charge fraction η=0.5 (thus $N_{1}=0$); (ii) a block copolymer with charge fraction η=0.5 (thus $N_{1}=49$); and (iii) a fully charged homopolymer with charge fraction η=1.0 (thus $N_{1}=99$). The valency of the counterions was varied from $Z_{C}=+1$ to $Z_{C}=+3$, and as this work is based on a coarse-grained model, it is possible to have fractional charges. To demonstrate this point, we included a case with $Z_{C}=+1.5$. Note that the case for η=1.0 and $Z_{C}=+1$ as well as those for η=1.0 and $Z_{C}=+2$ have been studied by Zhang et al. [44], and our results shown in Figure 5 agree quantitatively with theirs.

As shown in Figure 6, increasing the valence of the counterions expands the phaseseparated region and thus promotes phase separation. When the valence of the counterions is fixed, increasing the charge fraction results in two effects: (i) enhancing the electrostatic contribution (which promotes phase separation) and (ii) enhancing the contribution from translational entropy, as from charge neutrality, a higher charge fraction requires more counterions to maintain charge neutrality. If the valence of the counterion is large enough, e.g., $Z_{C}=3$, fewer counterions are needed to maintain charge neutrality, and therefore increasing the charge fraction always promotes phase separation.

In the case of liquid water at 1 atm, we have $\ell_{B}\in \left[7.0\;Å,8.0\;Å\right]$ (see Figure 3), and with σ=4.0Å, we have $\ell_{B}/\sigma \in \left[1.75,2.0\right]$. This means that aqueous solutions of the polymers investigated in Figure 5 with monovalent counterions will be in a single phase (no phase separation), as the critical Bjerrum length for phase separation is well above the accessible range for liquid water. However, as the counterion valency increases, phase separation could take place, as shown in Figure 6 for the cases with $Z_{C}=3$.

### 3.2. Effect of Chain Length and Charge Fraction

The critical point on the binodal curve of a salt-free solution can be fully characterized by a critical Bjerrum length $\ell_{B,c}$ (Y-coordinate) and a critical polymer segmental concentration $\rho_{p,c}$ (X-coordinate). This section addresses the question of how the critical point is affected by the polymer chain length and its charge fraction.

**Figure 7:**
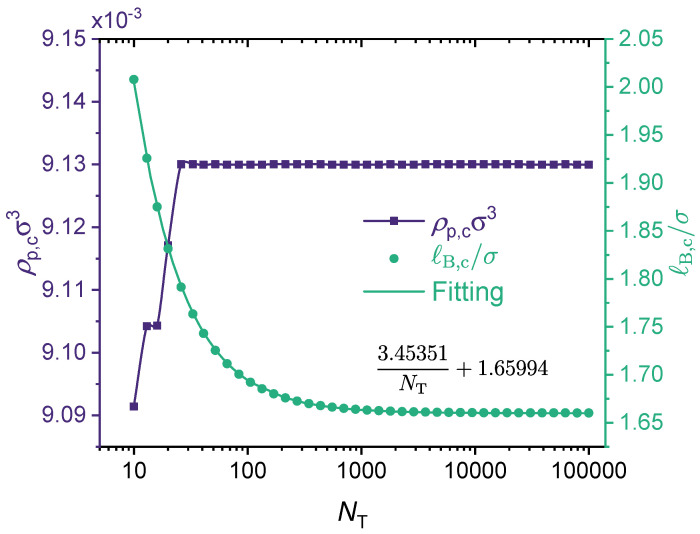
Effect of chain length: concentration of polymer $\rho_{p,c}$ (left y-axis) and Bjerrum length $\ell_{B,c}$ (right y-axis) of critical points in the binodal curves from the GUI App presented in this work. The values for the following parameters were fixed: $\epsilon_{AC}=\epsilon_{A}=\epsilon_{B}=0$, $Z_{A}=-1$, $Z_{C}=+2$, η=1. Values for $N_{T}$ is were varied, therefore, $N_{1}=N_{T}\eta -1$ was varied. Symbols are numerical results.

Figure 7 presents the effect of the polymer chain length parameter $N_{T}$ on the critical point for a fully charged polymer chain. As shown in the figure, the critical polymer concentration ($\rho_{p,c}$) is not sensitive to the chain length, although for small $N_{T}$ values it increases slightly with the increase of $N_{T}$. In comparison, the critical Bjerrum length $\ell_{B,c}$ decreases monotonically with the increase of $N_{T}$. The underlying physics is that a larger $N_{T}$ results in a lower translational entropy, which promotes phase separation.

As noted by Zhang et al. [44], previous theories [42,122,123] and Monte Carlo simulation [124] suggest that the critical concentration $\rho_{p,c}$ remains finite and is nearly independent of the chain length when $N_{T}>100$ because of the translational entropy of free counterions, while the critical Bjerrum length $\ell_{B,c}$ decreases slightly with increasing chain length due to both the smaller chain translational entropy and the stronger electrostatic correlation. Our results (see Figure 7) are fully consistent with these earlier findings. The results shown in Figure 7 correspond to a fully charged (charge fraction η=1) linear polymer with monovalent monomeric units ($Z_{A}=-1$) and divalent counterions ($Z_{C}=+2$). There is a very interesting feature in Figure 7 that has not been discussed in earlier studies. That is, while it is fair to say that the critical Bjerrum length decreases only slightly with increasing chain length, $\ell_{B,c}\approx 8.0\;Å$ for $N_{T}=10$, and $\ell_{B,c}\approx 6.65\;Å$ for $N_{T}>10^{4}$, this range of $\ell_{B,c}$ happens to overlap with the accessible Bjerrum length values for liquid water at 1 atm (see Figure 3). Recall that for water at room temperature (T≈293K), $\epsilon_{r}\approx 80$, meaning that $\ell_{B}\approx 7.1\;Å$. Therefore, if the chain is sufficiently short, phase separation may not take place because its critical Bjerrum length is above the Bjerrum length of the solvent. However, if the chain is long enough, then according to the results shown in Figure 7, phase separation can be expected because its critical Bjerrum length is now below the Bjerrum length of the solvent. It is interesting to observe in Figure 7 that the dependence of $\ell_{B,c}$ on the chain length parameter $N_{T}$ can be fitted well by an expression with the form

(30) $$\ell_{B,c}/\sigma =aN_{T}^{-1}+b$$

That is, if one plots $\ell_{B,c}/\sigma$ against $1/N_{T}$, the results follow a straight line. To the best of our knowledge, this finding has not been reported in previous studies. A follow-up question is how versatile this $\ell_{B,c}∼N_{T}^{-1}$ dependence is. This question is addressed later in this subsection.

**Figure 8:**
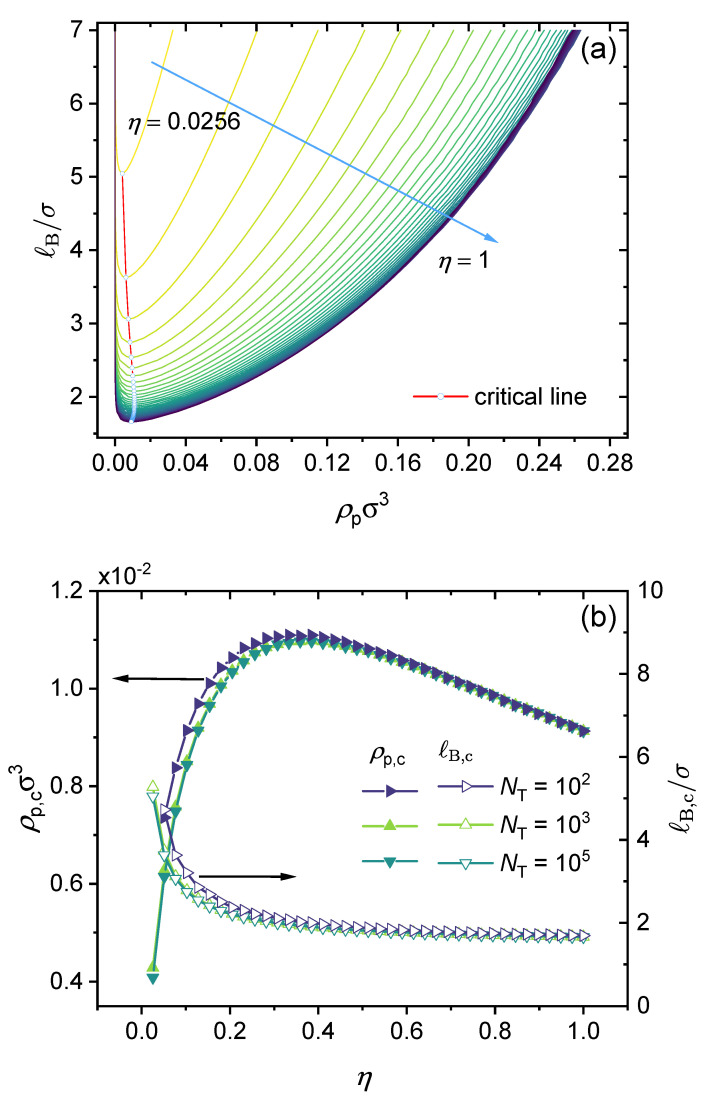
Effect of charge fraction: (**a**) Predicted binodal curves from the GUI App presented in this work. The values for the following parameters were fixed: $\epsilon_{AC}=\epsilon_{A}=\epsilon_{B}=0$, $Z_{A}=-1$, $Z_{C}=+2$, $N_{T}=10^{5}$, and $N_{1}=N_{T}\eta -1$ (block copolymer). (**b**) Concentration of polymer $\rho_{p,c}$ (left y-axis) and Bjerrum length $\ell_{B,c}$ (right y-axis) of critical points in the binodal curves versus η from 0 to 1 under different total lengths $N_{T}$ of polymer. The values of all parameters are the same as those used in (**a**) unless otherwise specified in the figure.

Figure 8 presents the effect of the polymer charge fraction on the binodal curve and the critical point. It is instructive to start from a simple general case, namely, partially charged polymers with divalent counterions only without adding salt and with no dispersion interactions or other specific interactions. In Figure 8a, we consider a realistic polymer chain length $N_{T}=10^{5}$ and an η varied from 0.0256 to 1 (fully charged). The $N_{1}$ parameter was set to be $N_{1}=N_{T}\eta -1$, which corresponds to the case of a block copolymer. Note that lowering the charge fraction reduces the effect of electrostatic interactions. As the results show, increasing η lowers the binodal curves monotonously. In the beginning, increasing η sharply enhances the phase separation, and the effect becomes milder as η approaches 1 (fully charged). In Figure 8b, it can be seen that while the critical Bjerrum length $\ell_{B,c}$ decreases monotonically as the charge fraction η increases, the dependence of the critical polymer concentration ($\rho_{p,c}$) on the polymer charge fraction (η) is not monotonic, and it first increases with the increase of η, reaches a maximum at η≈0.4 (to be more exact, η≈0.385), and then decreases with the further increase of η to 1 (fully charged). This non-monotonic behavior may be understood as a competition between electrostatic interaction and translational entropy. At a low charge fraction, a higher concentration of charged polymer is required to increase the electrostatic interaction. When the charge fraction (η) reaches approximately 0.385, the critical polymer concentration ($\rho_{p,c}$) reaches its maximum, and the critical Bjerrum length ($\ell_{B,c}$) becomes insensitive to the charge fraction. When η⪆0.385, which may be referred to as the critical charge fraction, increasing the charge fraction linearly decreases the critical concentration, with a slope of approximately −0.0035 (see Figure 8).

**Figure 9:**
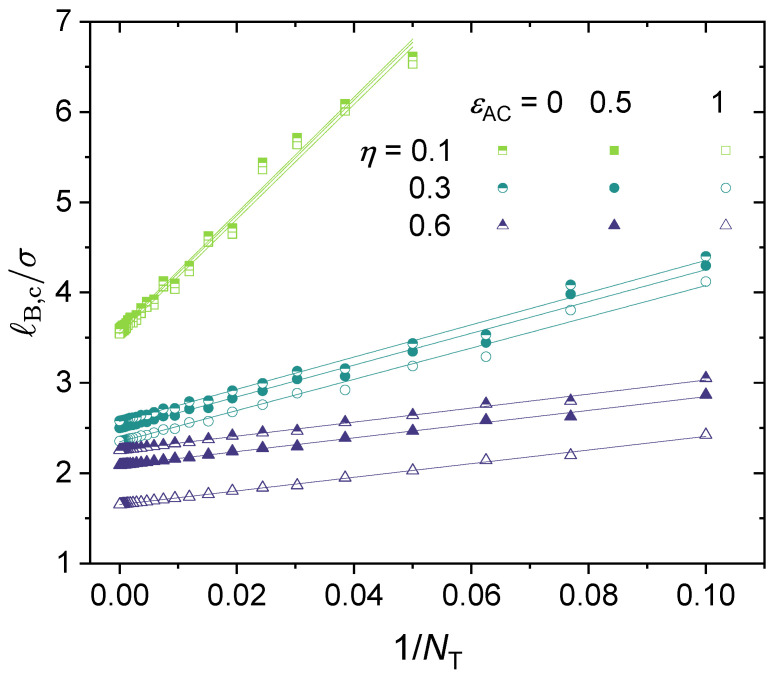
The scaling relationship: $\ell_{B,c}∼N_{T}^{-1}$. The values for the following parameters were fixed: $\epsilon_{A}=\epsilon_{B}=0$, $Z_{A}=-1$ and $Z_{C}=+2$. Values for $N_{T}$ is were varied, therefore $N_{1}=1/3N_{T}\eta -1$ was varied as well.

As mentioned in Figure 7, for a fully-charged polymer chain, the critical Bjerrum length scales as $\ell_{B,c}∼N_{T}^{-1}$ (see Equation (30)). To our knowledge, this is the first time such a scaling relation between $\ell_{B,c}$ and $N_{T}$ has been reported, though previous studies have noted that when $N_{T}$ is large, the phase separation is not sensitive to $N_{T}$ [44]. Figure 7 presents the results only for a fully charged polymer without any specific interactions. We investigate whether this scaling relationship can be generalized to all situations, i.e., for any values of η, $N_{1}$, *k*, and even ϵ>0. In Figure 9, we present results for nine cases under different $\epsilon_{AC}$ and η values to show that such a scaling dependence is followed for all these cases, and hence is quite general. For lower charge fractions, the phase separation is more sensitive to $N_{T}$. Note that the scaling law is followed, only with a larger slope (i.e., higher *a* in Equation (30)). Moreover, it seems that varying $\epsilon_{\mathrm{AC}}$ mainly affects the intercept *b*, and has little influence on the slope (see Figure 9).

**Figure 10:**
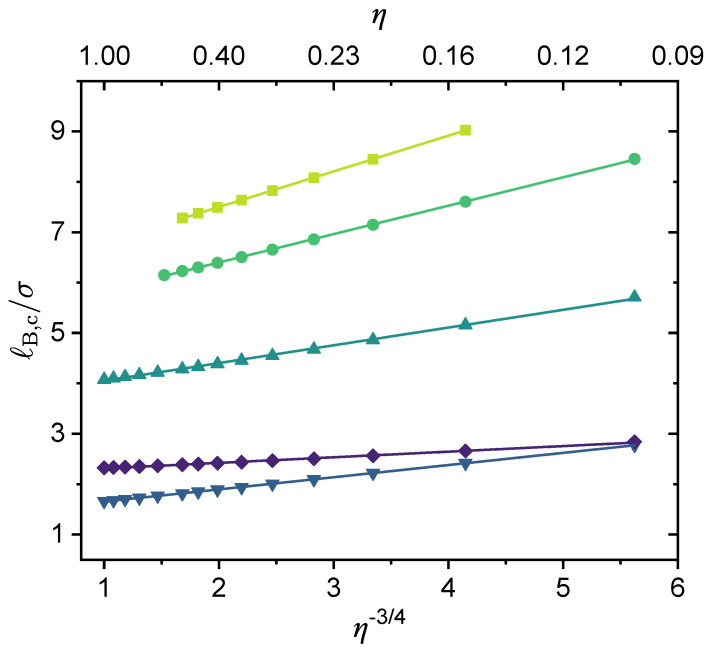
The scaling relationship: $\ell_{B,c}∼\eta^{-3/4}$. The values for the following parameters were fixed: $N_{T}=10^{4}$, $N_{1}=kN_{T}\eta -1$, and $\epsilon_{A}=\epsilon_{B}=\epsilon_{\mathrm{AC}}=0$. Values for η is were varied, and therefore values for the $N_{1}$-parameter were varied as well. From top to bottom: (i) $N_{1}=0$ (k→0), $Z_{p}=-1$, $Z_{C}=+1$; (ii) k=1/4, $Z_{p}=-1$, $Z_{C}=+1$; (iii) k=1, $Z_{p}=-1$, $Z_{C}=+1$; (iv) k=1, $Z_{p}=-2$, $Z_{C}=+1$; (v) $N_{1}=0$, $Z_{p}=-1$, $Z_{C}=+2$.

Following a similar idea, we have investigated whether there exists a simple scaling relationship between critical Bjerrum length ($\ell_{B,c}$) and the polymer charge fraction (η). As shown in Figure 10, it can be seen that in the absence of specific interactions ($\epsilon_{A}=\epsilon_{B}=\epsilon_{\mathrm{AC}}=0$), the critical Bjerrum length shows a linear dependence on $\eta^{-3/4}$ for $N_{T}\geq 10^{4}$, i.e., $\ell_{B,c}∼\eta^{-3/4}$. When $N_{T}$ is small, e.g., when $N_{T}=100$, $\ell_{B,c}∼\eta^{-0.922}$. When varying η values from 0.01 to 1, the scaling law of $\ell_{B,c}∼\eta^{-3/4}$ is followed for a broad range of η values. Hence, the larger the charge fraction, the smaller the critical Bjerrum length (see Figure 8b). In Figure 10, we introduce a *k*-parameter to describe the monomer sequence distribution and define the $N_{1}$-parameter as $N_{1}=kN_{T}\eta -1$. Here, the *k*-parameter has a maximum value of 1, which is for the case of a block copolymer. As the results show, the larger the *k* value, the smaller the critical Bjerrum length. The effect of the $N_{1}$-parameter was discussed in our recent work [91]; increasing $N_{1}$ without changing other parameters increases the electrostatic contribution to the excess Helmholtz free energy density, and hence promotes phase separation. We varied the valency of the charged groups; as is shown in Figure 10, increasing the valency of the charged groups of the polymer or the counterions lowers the critical Bjerrum length.

Notice that once the *k*-parameter, k∈(0,1], is provided, the available charge fraction cannot exceed its maximum of $\eta_{max}=1/\left(2-k\right)$ (which can be derived from Equation (29)). Therefore, for k→0, k=1/4, and k=1, the corresponding $\eta_{max}$ values ae 0.5, 0.57, and 1, respectively. This explains why for the two curves at the top we cannot reach higher η values than those shown in Figure 10.

### 3.3. Effect of Local Short-Range Interactions

**Figure 11:**
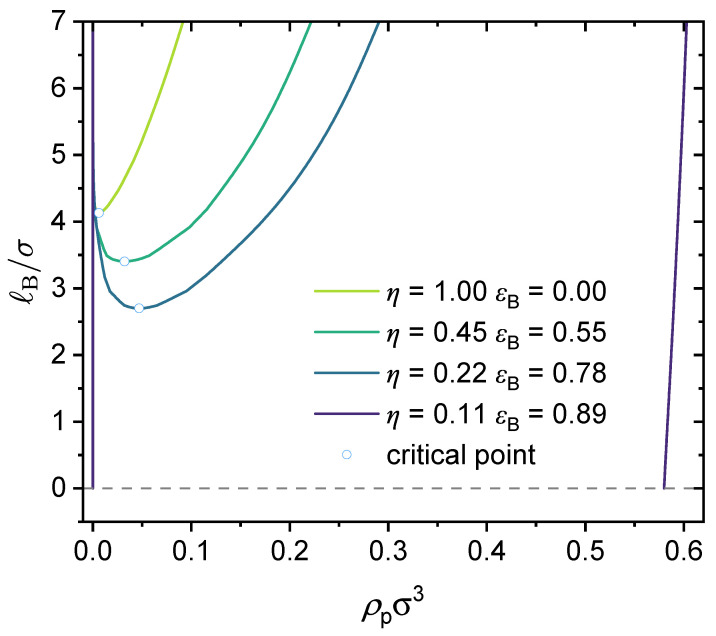
Predicted binodal curves from the GUI App presented in this work. The values for the following parameters were fixed: $\epsilon_{A}=0$, $\epsilon_{AC}=0$, $N_{T}=100$, $Z_{C}=+1$ and $Z_{A}=-1$. Values for the charge fraction η and the interaction strength between the neutral segments of the polymer $\epsilon_{B}$ were varied. As η was varied, the $N_{1}$-parameter, $N_{1}=N_{T}\eta -1$ (block copolymer), was varied as well. The dashed line shows the limiting value of $\ell_{B}/\sigma =0$.

**Figure 12:**
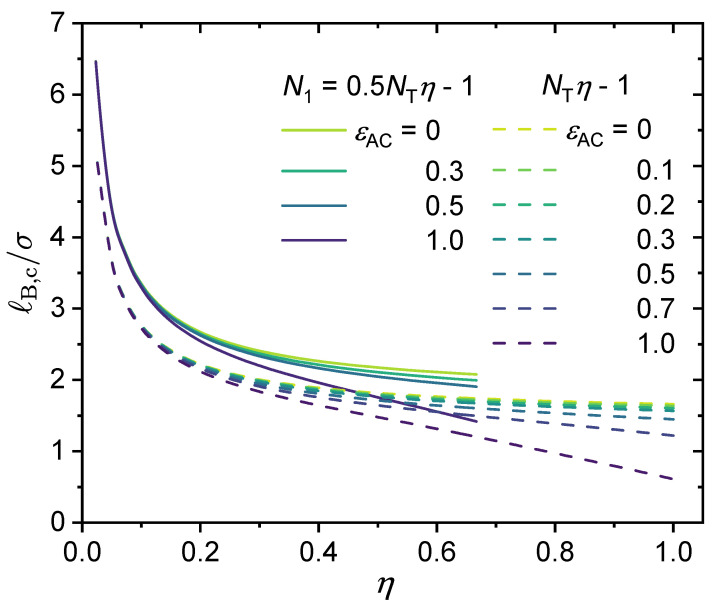
Bjerrum length of critical points $\ell_{B,c}$ in the binodal curves. The values for the following parameters were fixed: $\epsilon_{A}=\epsilon_{B}=0$, $Z_{A}=-1$, $Z_{C}=-2$, $N_{T}=10^{5}$. Values for η were varied under different $\epsilon_{AC}$ and $N_{1}$ values.

**Figure 13:**
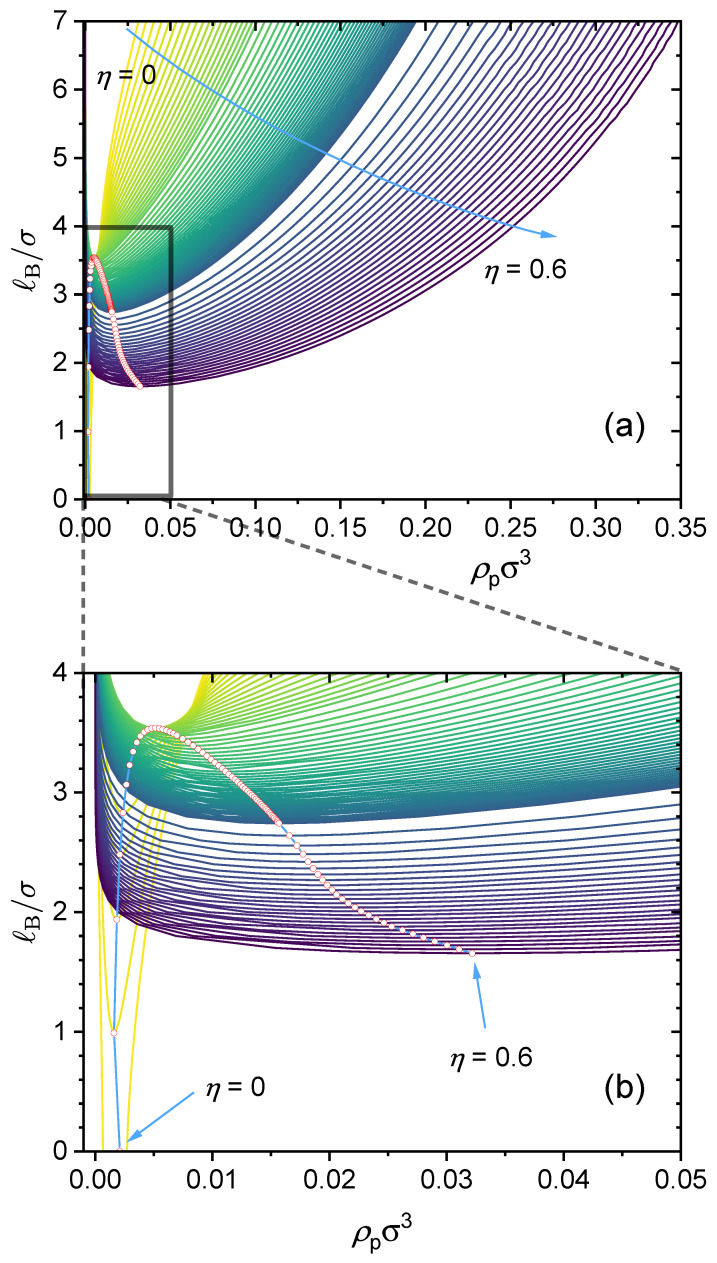
(**a**) Predicted binodal curves from the GUI App presented in this work. The values for the following parameters were fixed: $\epsilon_{AC}=1$, $\epsilon_{B}=0.2$, $\epsilon_{A}=0$, $Z_{A}=-1$, $Z_{C}=-2$, $N_{T}=10^{5}$. Values for η were varied, therefore, $N_{1}=1/3N_{T}\eta -1$ was varied as well. The blue arrow indicates the direction of increasing charged fraction (η from 0 to 0.6). The red hollow circles are the critical points. (**b**) The subfigure at the bottom is a zoomed-in view showing a closer look at the binodal curves for the range of $\ell_{B}/\sigma \in \left[0,\;4\right]$ and $\rho_{p}\sigma^{3}\in \left[0,\;0.05\right]$.

**Figure 14:**
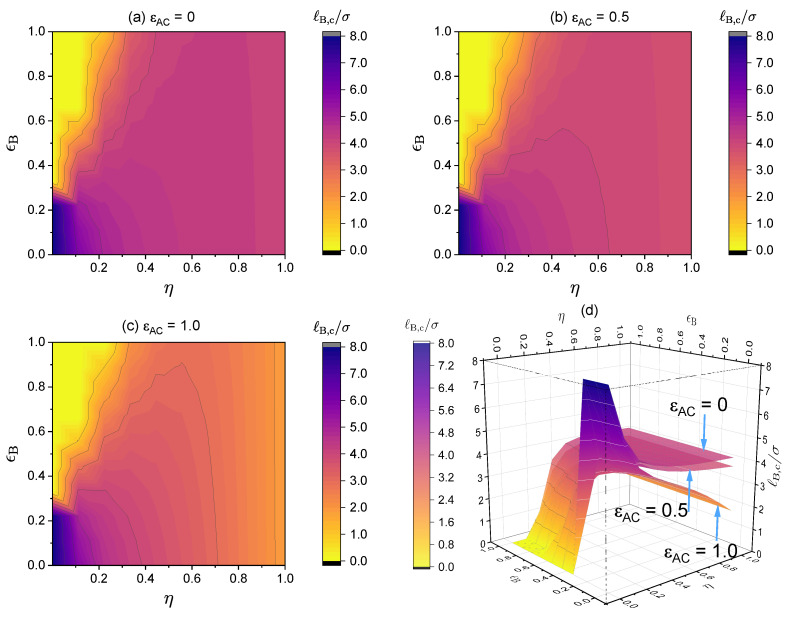
Critical Bjerrum length ($\ell_{B,c}/\sigma$) for the critical points in the binodal curves. The values for the following parameters were fixed: $\epsilon_{A}=0$, $N_{T}=100$, $Z_{C}=+1$ and $Z_{A}=-1$ and (**a**) $\epsilon_{AC}=0$, (**b**) $\epsilon_{AC}=0.5$ and (**c**) $\epsilon_{AC}=1.0$. Values for η and $\epsilon_{B}$ were varied, therefore, $N_{1}=N_{T}\eta -1N_{1}$ was varied as well. (**d**) The 3D surfaces for (**a**–**c**). Notice that the maximum of $\ell_{B}/\sigma$ shown here is $\ell_{B}/\sigma =8$.

Here, we investigate in detail the effects of short-range specific interactions on the binodal curves and the critical points. Figure 11 presents the effect of specific interactions between the neural segments of the polymer, where we varied the interaction strength $\epsilon_{B}$ and the charge fraction. Notice that for the cases of η=0.11 and $\epsilon_{B}=0.89$, it is predicted that the system is always in a phase-separated situation, as its phase separation is mainly driven by the dispersion interaction $\epsilon_{B}$, which is assumed to be athermal ($U_{disp}∼k_{B}T$; see Equation (5)) in the present model. Figure 12 presents the effect of the specific interactions between the charged segments of the polymer and the counterions on the relationship between the critical Bjerrum length and the charge fraction, where we varied the interaction strength $\epsilon_{AC}$ and the monomer sequence distribution (k=0.5 vs. k=1). Figure 13 presents the binodal curves for a series of partially charged polymers in the presence of specific interactions described by $\epsilon_{AC}=1.0$ and $\epsilon_{B}=0.2$, where we have varied the charge fraction from η=0 to η=0.6. In Figure 14, we show how the critical Bjerrum length depends on the charge fraction (η) and the specific interactions (strength parameter $\epsilon_{B}$) between the neural segments of the polymer for different $\epsilon_{AC}$ values. All these results were obtained by the computational App presented in Figure 4, and are reprocessed and assembled here for better presentation of the results.

If there is no specific interaction present, decreasing the polymer charge fraction increases the critical Bjerrum length, as shown earlier in Figure 10. However, after we include an attractive short-range interaction between the neural segments of the polymer, it is possible that partially charged polymers may have an even lower critical Bjerrum length than their fully charged case (see Figure 11). The lower the charge fraction, the more interaction pairs between the neutral segments; hence, when the interaction strength is strong enough, such specific interactions may become the dominating force for phase separation.

When increasing $\epsilon_{AC}$, the strength of the short-range interaction between the charged segments of the polymer and the counterions further lowers the critical Bjerrum length in comparison to cases in which such interactions are not present. As shown in Figure 12, such an effect becomes more pronounced as the charge fraction increases, as there are more such interacting pairs compared to the case of polymers with a lower charge fraction. Moreover, the results shown in Figure 12 reinforce our earlier findings that (i) the larger the charge fraction, the lower the critical Bjerrum length and (ii) the larger the $N_{1}$-parameter (or equivalently, the *k*-parameter), the lower the critical Bjerrum length. We may now add (iii): the larger the $\epsilon_{AC}$-parameter, the lower the critical Bjerrum length.

As an example. Figure 13 presents a case study where two types of specific interactions are present: $\epsilon_{AC}=1.0$ and $\epsilon_{B}=0.2$. The binodal curves are shown for various charge fractions in the range of [0,0.6]. As seen from the dependence of the critical point on the charge fraction, when η<0.024, phase separation is mainly determined by the attractive short-range interaction between the neural segments of the polymer, $\epsilon_{B}$, and therefore, as η increases, the critical Bjerrum length increases and it becomes more difficult for phase separation to occur. When η>0.024, phase separation is mainly determined by electrostatic interaction and $\epsilon_{AC}$, and therefore the critical Bjerrum length decreases as η increases. Notice that the competition between the electrostatic interaction and both $\epsilon_{AC}$-dominated phase separation and $\epsilon_{B}$-dominated phase separation depends on the respective values of $\epsilon_{AC}$ and $\epsilon_{B}$. Therefore, the critical charge fraction (η) separating those two regimes depends on the values of $\epsilon_{AC}$ and $\epsilon_{B}$ used in our calculations. This is investigated further in Figure 14.

Figure 14 presents density plots and a 3D plot to show how the critical Bjerrum length depends on the charge fraction (η) and specific interactions between the neutral segments of the polymer, characterized by a strength parameter $\epsilon_{B}$, for three different $\epsilon_{AC}$ values: (a) $\epsilon_{AC}=0$, (b) $\epsilon_{AC}=0.5$, and (c) $\epsilon_{AC}=1.0$. The existence of the two regimes, namely, electrostatic interaction along with $\epsilon_{AC}$-dominated phase separation and $\epsilon_{B}$-dominated phase separation, is apparent from the clear non-monotonic dependence of the critical Bjerrum length on the polymer charge fraction with a sufficiently large $\epsilon_{B}$ value. Note that we consider monovalent counterions in Figure 14. Changing the monovalent counterions to divalent or multivalent counterions brings the critical Bjerrum length to lower values, as demonstrated earlier in Figure 6.

As noted earlier, in the case of liquid water at 1 atm, we have $\ell_{B}\in \left[7.0\;Å,8.0\;Å\right]$ (see Figure 3), and with σ=4.0Å, we have $\ell_{B}/\sigma \in \left[1.75,2.0\right]$. In the absence of specific interactions ($\epsilon_{AC}=\epsilon_{B}=0$), aqueous solutions of the polymers investigated in Figure 14 with monovalent counterions will be in a single phase (no phase separation) because the critical Bjerrum length for phase separation is well above the accessible range for liquid water. However, in the presence of specific interactions between the neural segments of the polymer, phase separation could take place for partially charged polymers if $\epsilon_{B}$ becomes sufficiently large.

## 4. Conclusions

In the present work, the phase behavior of partially charged ion-containing polymers in polar solvents is studied by further developing a liquid state theory with local short-range interactions. This work is based on the LS theory previously developed for fully-charged polyelectrolyte solutions. Specific interactions between charged groups of the polymer and counterions, specific interactions between neutral segments of the polymer, and specific interactions between charged segments of the polymer are incorporated into the liquid state theory by an extra Helmholtz free energy from the PC-SAFT model. The influence of the sequence structure of the partially charged polymer is modeled by the number of connections (the $N_{1}$-parameter) between bonded segments. The effects of chain length ($N_{T}$), charge fraction (η), valencies of charged groups and counterions ($Z_{A}$ and $Z_{C}$), and specific short-range interactions ($\epsilon_{A}$, $\epsilon_{AC}$, and $\epsilon_{B}$) are explored.

Concerning the effect of polymer chain length and charge fraction, we report for the first time that (i) the critical Bjerrum length ($\ell_{B,c}$) decreases as the chain length increases and follows a scaling relation of $\ell_{B,c}∼\eta^{-1}$, and (ii) for $N_{T}>10^{4}$ and in the absence of specific interactions, the critical Bjerrum length ($\ell_{B,c}$) decreases as the charge fraction increases and follows a scaling relation of $\ell_{B,c}∼\eta^{-3/4}$. The linear dependence of the critical Bjerrum length on $N_{T}^{-1}$, i.e., $\ell_{B,c}/\sigma =aN_{T}^{-1}+b$, where *a* and *b* are fitting parameters of the polymer following $N_{T}^{-1}$, is rather general and holds for a variety of $N_{T}$, η, $Z_{A}$ and $Z_{C}$, the $N_{1}$-parameter, and strengths of the specific interactions $\epsilon_{A}$, $\epsilon_{AC}$, and $\epsilon_{B}$. These scaling relations allow for simple and useful predictions of the critical Bjerrum length for solution phase behavior.

In addition to the two scaling relations found in the present study, the results presented in this work elucidate in detail the influence of the valencies of charged groups and counterions, monomer sequence distribution (characterized by the $N_{1}$-parameter, and specific interactions between the neural segments of the polymer (as a primitive model for hydrophobic interactions) and between the charged groups of the polymer and counterions (as a primitive model for ion-binding interactions). Increasing the valence of the counterions reduces the critical Bjerrum length and expands the phase-separated region, thus promoting phase separation. Increasing the $N_{1}$-parameter without changing other parameters promotes phase separation; therefore, for partially charged polymers of a given charge fraction, the block-type of monomer sequence distribution is more prone to phase separation. Increasing the strength of specific interactions between the charged groups of the polymer and counterions promotes phase separation, and the larger the $\epsilon_{AC}$-parameter, the lower the critical Bjerrum length.

If there is no specific interaction present between the neural segments of the polymer, decreasing the polymer charge fraction monotonically increases the critical Bjerrum length. However, when including an attractive short-range interaction between the neural segments of the polymer, it is possible that partially charged polymers may have an even lower critical Bjerrum length than their fully charged case. The lower the charge fraction, the more interaction pairs between the neutral segments; hence, when the interaction strength is strong enough, such specific interactions may become the dominating force for phase separation. For partially charged polymers with specific interactions between neutral segments, there may exist two regimes in their phase behavior, namely, electrostatic interaction and $\epsilon_{AC}$-dominated phase separation and $\epsilon_{B}$-dominated phase separation. In such cases, the critical Bjerrum length may show a non-monotonic dependence on the polymer charge fraction, in which it first increases with increasing charge fraction in the $\epsilon_{B}$-dominated phase separation regime, then decreases with further increasing charge fraction in the electrostatic interaction and $\epsilon_{AC}$-dominated phase separation regime.

To facilitate easy access to the theoretical model and numerical results presented in this work, a computational App for the salt-free case is presented and provided in the Supplementary Materials. This App allows easy computation of the binodal curve and critical point by specifying values for the relevant model parameters.

For future work, it remains necessary to compare the theoretical predictions of this work with results from experiments or computer simulations. Our results on the effect of chain length agree with earlier findings on the effect of chain length on the phase behavior of fully-charged polyelectrolytes, and our results on the qualitative effect of charge fraction and that of the specific interactions are more or less expected. However, on a more quantitative level, we are not aware of any existing experimental or computer simulation studies that address these effects. We encourage research groups with such expertise to investigate the two scaling relations reported in this work.

In addition, it would be interesting to look into the structural details of the phase behavior of ion-containing polymers. The present work does not provide such structural information, as it is based on a mean-field formulation. It is possible to extend the work to a DFT formulation, similar to that of Xu et al. [63], who investigated the structural and responsive properties of grafted polyanion chains subjected to the effects of dispersion interaction and salt. It is foreseeable that by extending the present research to a DFT formulation, it could be possible to explore the rich structural properties of ion-containing polymers with dispersion interactions and end-tethered weak ion-containing polymers at interfaces. The study of weak ion-containing polymers that are confined in an interface is a challenging field, although a very interesting one [125].

## Figures and Tables

**Table 1:** Summary of the different species considered in this work. A = Charged segments of the polymer; B = Neutral segments of the polymer, C = Counterions of the charged segments of the polymer and salt co-ions, D = Co-ions from the added salt (assumed to be C+D).

Component	A	B	C	D
Number density	$\rho_{A}$	$\rho_{B}$	$\rho_{C}$	$\rho_{D}$
Valence	$Z_{A}$	$Z_{B}=0$	$Z_{C}$	$Z_{D}$

**Table 2:** Input parameters used in the GUI App. For the salt-free polymer solution, there are three types of beads (see Figure 1): Type A = Charged segments of the polymer; Type B = Neutral segments of the polymer; Type C = Counterions. The strength parameter for the dispersion interaction is introduced in Equation (5).

Notions	Definition
$\ell_{B}$ max	Upper bound of the range of the Bjerrum length
$\ell_{B}$ min	Lower bound of the range of the Bjerrum length
$\ell_{B}$ step	Step length (bin size) of the Bjerrum length
$N_{T}$	Total number of (A+B) segments of the polymer chain
$N_{1}$	Number of bond connections between charged segments (A)
η	Charge fraction of the polymer chain
$\epsilon_{AC}$	Strength of dispersion interaction between A and C
$\epsilon_{A}$	Strength of dispersion interaction between A and A
$\epsilon_{B}$	Strength of dispersion interaction between B and B
$Z_{A}$	Valence of individual ionized groups of the polymer
$Z_{C}$	Valence of counterions

## Data Availability

The Matlab codes that were used to produce the results shown in this work are available in the Supplementary Materials of this work. All other data can be made available on request.

## Supplementary Materials

The following are available at: https://www.mdpi.com/article/10.3390/polym14204421/s1; The Matlab codes that were used to produce the results shown in this work are available in the Supplementary Materials of this work.

## Abbreviations

The following abbreviations are used in this work:

BMCSL	Boublík–Mansoori–Carnahan–Starling–Leland
cDFT	classical Density Functional Theory
GUI App	Graphical User Interface Application
HPAM	partially hydrolyzed polyacrylamide
IUPAC	International Union of Pure and Applied Chemistry
LCST	Lower Critical Solution Temperature
LS	Liquid State
MSA	Mean-Spherical Approximation
PAA	Poly(acrylic acid)
PCEs	Polycarboxylate (ether/ester)-based Superplasticizers
PC-SAFT	Perturbed-Chain Statistical Associating Fluid Theory
PMAA	Poly(methacrylic acid)
TPT1	first-order thermodynamic perturbation theory
TRUE	Transparent, Reproducible, Usable by others, and Extensible
